# Re: A Meta-Analysis of the Clinical Use of Curcumin for Irritable Bowel Syndrome

**DOI:** 10.3390/jcm8111885

**Published:** 2019-11-06

**Authors:** Laura Appleton, Andrew S. Day

**Affiliations:** Department of Paediatrics, University of Otago Christchurch, 8140 Christchurch, New Zealand; laura.appleton@otago.ac.nz

We read with interest the article by Ng et al. [1] on the use of curcumin in irritable bowel syndrome (IBS). This work systematically reviewed five randomised-controlled trials, and included three for meta-analysis (a total of 326 subjects). The authors concluded that while safe, well tolerated, and beneficial in reducing IBS symptoms, these results were not statistically significant based on the limited evidence available. 

IBS is a complex, functional gastrointestinal disorder typically characterized by chronic abdominal pain, altered bowel habit, and abdominal bloat [2]. Despite being one of the most common GI disorders, the underlying pathophysiology remains unclear. Traditionally, the aetiology of IBS has focused on altered gut motility, brain–gut interactions, visceral hypersensitivity, and psychosocial distress [3]. More recent evidence implicates a range of other factors including changes to gut immune activation, intestinal permeability, alterations in faecal flora, and bacterial dysbiosis [4,5]. The latter two factors are particularly evident in the development of post-infectious IBS [6]. Studies have also highlighted the persistence of low-grade mucosal inflammation at the microscopic and molecular level in patients with IBS, with increased recruitment of enteroendocrine cells [7]. 

Reflecting the various aetiologic factors and the varied clinical presentations (for example, diarrhoea predominant versus constipation predominant IBS), many management options have been considered for IBS. One recent advance in management of IBS has been the use of a diet low in fermentable oligo-, di-, monosaccharides, and Polyols (FODMAPs), which can lead to improvement in symptoms [8,9,10,11]. However, this dietary intervention is not universally beneficial. Further, given the lack of definitive therapies for IBS, there is much interest in new possible interventions. 

Curcumin, derived from turmeric root (*Curcuma longa*), is known to have numerous actions within the body, including anti-inflammatory, antitumor, and antioxidant effects [12,13,14,15,16,17,18,19,20]. It is believed to modulate cell signalling molecules such as pro-inflammatory cytokines, apoptotic proteins, C-reactive protein, and also inhibits the nuclear factor (NF)-κB intracellular signal transduction pathway [21]. 

Curcumin has been investigated for numerous human ailments such as arthritis, Alzheimer’s, inflammatory bowel disease, and cancer [22,23,24,25,26,27,28,29,30,31]. However, low bioavailability due to poor absorption, rapid metabolism, and rapid systemic elimination potentially limit its therapeutic efficacy [32] requiring either high doses or the need for adjuvants or additives. By way of example, the absorption of curcumin is increased by up to 2000% when combined with piperine [33]. 

Whilst this meta-analysis did not find curcumin to have a statistically significant effect on reducing IBS symptoms, the included studies varied widely, with likely implications on the findings arising [1]. For example, a variety of doses were seen in the included studies; ranging from 200 to 8000 mg daily, for periods of 3 weeks to 6 months. 

Since the publication of the meta-analysis, there has been one further report evaluating curcumin in patients with IBS [34]. This open-label pilot study evaluated the use of curcumin in combination with fennel oil for two months in patients with IBS seen in a primary health care setting in Belgium. The results of this work indicated improved quality of life and reduced symptoms following treatment. Although this study was a real-life study and included 211 subjects, it did not include a randomised placebo-controlled arm.

In conclusion, while curcumin appears to have potential clinical benefits, especially in the setting of IBS, the definitive role of this intervention is not confirmed. As highlighted by the authors of the meta-analysis, further studies are required. These should be based upon a clear consensus for a consistent therapeutic dose and dosing regimen, whether that be via optimisation of the daily dose or by using an adjuvant to increase bioavailability of curcumin. 
